# Editorial Comment to Expression of tertiary lymphoid structure in deferred cytoreductive nephrectomy of metastatic renal cell carcinoma treated with nivolumab plus ipilimumab

**DOI:** 10.1002/iju5.12360

**Published:** 2021-08-17

**Authors:** Takeshi Yuasa

**Affiliations:** ^1^ Department of Urology Cancer Institute Hospital Japanese Foundation for Cancer Research Tokyo Japan

The combination of the immune checkpoint inhibitors (ICIs), nivolumab (Optivo; Ono Pharma/Bristol Myers Squib), an antibody that targets programmed death‐1, plus ipilimumab (Yervoy; Bristol Myers Squib), an antibody that targets cytotoxic T‐lymphocyte‐associated antigen‐4, has been rapidly introduced as first‐line therapy for the treatment of metastatic renal cell cancer (RCC) within the International Metastatic Renal Cell Cancer Database Consortium (IMDC) intermediate/poor category.^1^, ^2^ The paradigm of medical treatment for patients with metastatic RCC has been dramatically changing. Therefore, biomarkers to predict the response and prognosis of ICI therapy are urgently needed. Amongst such biomarkers, tertiary lymphoid structures (TLSs) are ectopic lymphoid organs that develop in non‐lymphoid tissues.^3^, ^4^ Currently, the presence of TLSs is considered to be one of the most important predictive and prognostic factors in immune therapy.^3^, ^4^

In this issue of *IJU Case Reports,* Sazuka *et␣al*. reported two patients with metastatic renal cell carcinoma, who demonstrated a complete response after combined nivolumab plus ipilimumab therapy followed by the deferred cytoreductive nephrectomy.^5^ At the initiation of the nivolumab plus ipilimumab therapy, both patients were in the IMDC poor‐risk category with multiple metastases.^5^ Because almost all metastatic sites disappeared following the ICIs combination, both patients underwent the deferred cytoreductive nephrectomy.^5^ Pathological analysis disclosed that there were no or only a few viable cancer cells present in the kidney.^5^ Instead, there were several TLSs near the necrotic tumor and a large number of lymphocytes in both cases.^5^ Although they did not receive any treatment after the cytoreductive nephrectomy, they both survived with no evidence of disease.^5^

Accumulating evidence indicates that TLSs play a major role in controlling cancer cell invasion and metastasis.^3^, ^4^ TLSs are not formed prenatally and are not present under normal conditions. TLSs are found in chronically inflamed locations, which include persistent pathogen infection, autoimmune disorders, allograft rejection, and cancer.^3^, ^4^ Despite developing evidence that TLSs are intrinsic to the immune response in cancer, their application as not only therapeutic tools but also biomarkers is far from real‐world clinical practice.^3^, ^4^ Therefore, further investigation is necessary to utilize the finding of TLSs effectively and to obtain the clinical benefit for cancer patients.

## Conflict of interest

The author declares no conflict of interest.
